# Atrazine Inhalation Causes Neuroinflammation, Apoptosis and Accelerating Brain Aging

**DOI:** 10.3390/ijms22157938

**Published:** 2021-07-26

**Authors:** Tiziana Genovese, Rosalba Siracusa, Roberta Fusco, Ramona D’Amico, Daniela Impellizzeri, Alessio Filippo Peritore, Rosalia Crupi, Enrico Gugliandolo, Rossana Morabito, Salvatore Cuzzocrea, Angela Trovato Salinaro, Marika Cordaro, Rosanna Di Paola

**Affiliations:** 1 Department of Chemical, Biological, Pharmaceutical and Environmental Sciences, University of Messina, Viale Ferdinando Stagno D’Alcontres 31, 98166 Messina, Italy; tgenovese@unime.it (T.G.); rsiracusa@unime.it (R.S.); rfusco@unime.it (R.F.); rdamico@unime.it (R.D.); dimpellizzeri@unime.it (D.I.); aperitore@unime.it (A.F.P.); rmorabito@unime.it (R.M.); dipaolar@unime.it (R.D.P.); 2 Department of Veterinary Sciences, University of Messina, 98168 Messina, Italy; rcrupi@unime.it (R.C.); egugliandolo@unime.it (E.G.); 3 Department of Pharmacological and Physiological Science, Saint Louis University School of Medicine, Saint Louis, MO 63104, USA; 4 Department of Biomedical and Biotechnological Sciences, University of Catania, 95124 Catania, Italy; 5 Department of Biomedical, Dental and Morphological and Functional Imaging University of Messina, Via Consolare Valeria, 98125 Messina, Italy; marika.cordaro@unime.it

**Keywords:** atrazine, endocrine disruptor, oxidative stress, inflammation, brain alterations, aging

## Abstract

Background: exposure to environmental contaminants has been linked to an increased risk of neurological diseases and poor outcomes. Chemical name of Atrazine (ATR) is 6-chloro-*N*-ethyl-*N*′-(1-methylethyl)-1,3,5-triazine-2,4-diamine, and it is the most commonly used broad-spectrum herbicide in agricultural crops. Several studies have demonstrated that ATR has the potential to be harmful to the brain’s neuronal circuits. Until today nobody has explored the effect of ATR inhalation on young and aged mice. Methods: young and aged mice were subject to 25 mg of ATR in a vehicle made with saline and 10% of Dimethyl sulfoxide (DMSO) every day for 28 days. At the end of experiment different behavioral test were made and brain was collected. Results: exposure to ATR induced the same response in terms of behavioral alterations and motor and memory impairment in mice but in aged group was more marked. Additionally, in both young and aged mice ATR inhalations induced oxidative stress with impairment in physiological antioxidant response, lipid peroxidation, nuclear factor kappa-light-chain-enhancer of activated B cells (nf-κb) pathways activation with consequences of pro-inflammatory cytokines release and apoptosis. However, the older group was shown to be more sensitive to ATR inhalation. Conclusions: our results showed that aged mice were more susceptible compared to young mice to air pollutants exposure, put in place a minor physiologically response was seen when exposed to it.

## 1. Introduction

As the global average life expectancy rises, so does the amount of time available for prolonged exposure to harmful elements in the environment. As a result, the presence of low amounts of xenobiotic substances over time can have an effect on the aging process. Because the interaction of neurotoxic substances with the normal aging process can be slow and gradual, it might be difficult to detect [1].

The aging brain is marked by elevated levels of inflammatory markers [2,3,4,5]. This occurs even in the absence of foreign inflammatory stimuli, and is likely to reflect the accumulation of immune system responses to previous activation. Following the insult, these reactive activities may be maintained rather than distributed [6]. Different studies showed that after exposure to several pollutants, experimental animals show a significantly increase in cerebral inflammation suggesting a promotion of neurodegeneration [7,8,9,10]. This is probably due to the fact that the combination of such effects with natural aging may lead to the onset of Parkinsons and other neurodegenerative illnesses at an earlier age. Many molecules, even though their primary effect appears to be on other organs, can cause inflammatory changes in the nervous system, which can subsequently exacerbate the normal and corresponding changes that occur in the aging organism [1]. These changes could combine to over-activate the responses in the central nervous system (CNS), which is already experiencing modest age-related increases in intrinsic inflammation. In this approach, xenobiotics found in the environment might amplify the effects of processes already underway in the aging brain [1]. Exposure to environmental toxins such as pesticides may hasten the deterioration of the aged nervous system’s dopaminergic capacity. Bäckman et al. found that the harmful effects of dopaminergic toxins are compounded by the natural loss of dopaminergic neurons [11]. Toxicants can have more overt effects in the aged brain because the nervous system’s ability to endure toxic stimuli is already decreased.

Occupational or constant exposure to pesticides occurs during their production, transport, and storage or during user’s preparation and application as well as, during re-entry into treated fields, harvests, and equipment cleaning [12,13]. The majority of pesticides used in agriculture enter the body through the skin, followed by the respiratory and oral paths.

The chemical name for Atrazine (ATR) is 6-chloro-*N*-ethyl-*N*′-(1-methylethyl)-1,3,5-triazine-2,4-diamine, and it is the most commonly used broad-spectrum herbicide in agricultural crops such as corn, sorghum, and sugarcane [14]. Despite the fact that farm use of ATR is limited in the Europe Union (EU), it is still one of the most widely used pesticides in the world, with ATR being found in ground water in the United States and EU on a regular basis [14,15]. ATR has a half-life of 95–350 days and is resistant to degradation in fact, following application, it can be present in the particulate and vapor phases of the air, and it can travel up to 186 miles from the application site [16]. ATR can be broken down in the air by reacting with hydroxyl radicals [17]. ATR contamination has been linked to many different serious health issues including neurologic conditions, [18,19,20,21,22,23,24,25,26,27,28]. In particular, its well established that ATR is able to induce changes in the antioxidant response in brain of common carp, behavioral alterations and brain acetylcholinesterase deficits in zebrafish, brain alterations in GABA, glutamate and glutamine markers in the male albino rat and many others [29,30,31,32].

But, until today, nobody has explored the impact of ATR exposure on aging brain. Considering that ATR is released into the atmosphere as a result of its preparation, manufacturing, and disposal, and that it also enters the environment through the loss of applied herbicide until it enters the soil surface, as well as the particle distribution of ATR-containing dust, and considering that ATR volatilization after application to fields has been estimated to be up to 14% of the applied volume, it is critical to investigate the effects of air exposure on aging people. In particular, we supposed that these alterations were probably linked with the oxidative stress conditions following an imbalanced condition of physiological antioxidant ATR-induced [33,34,35,36,37,38]. Understanding the molecular basis of ATR-induced oxidative stress, apoptosis, and inflammation processes is critical for the development of therapeutic approaches to limit compromised brain decline. With this aims in our mind, we investigated for the first time the impact of ATR aerosol inhalation on young and aged mice.

## 2. Results

### 2.1. ATR Inhalation Induces Anxiety and Depression

**Figure 1:**
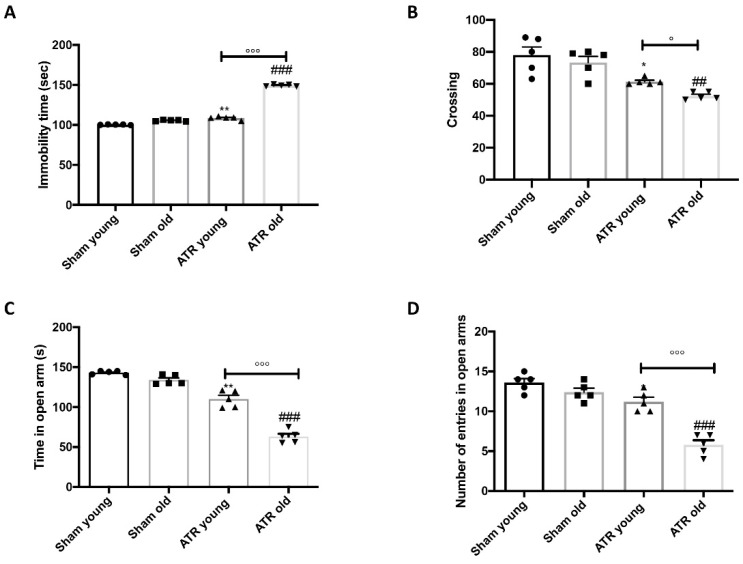
Effect of ATR inhalation on anxiety and depression. Forced Swim Test (**A**); Open Field test (**B**); Time in open arms (**C**) and number of entries in open arms (**D**) was recorded during Elevated Plus Maze test. During the behavioral tests we found that aged mice were more susceptibility compared to young to show anxiety and depression after ATR inhalation. Data is expressed as the mean ± SEM of *n* = 5 animals for each group. * *p* < 0.05 vs. Sham young; ** *p* < 0.01 vs. Sham young; ## *p* < 0.01 vs. Sham old; ### *p* < 0.001 vs. Sham old; ° *p* < 0.05 ATR old vs. ATR young; °°° *p* < 0.001 ATR old vs. ATR young.

To investigate the impact of ATR inhalation on anxiety and depression we performed different behavioral tests. Analysis of the data of Forced Swim (Figure 1A), Open Field (Figure 1B), and Elevated Plus Maze (Figure 1C,D) tests, revealed that after 28 days of ATR exposure, animals suffering of a very important anxiety and depression conditions compared to control group. In details, both young and aged mice showed a significantly increased anxiety and depression condition compared to the respectively control, but when we compared the data of aged mice to young mice, we found aged mice were more susceptibile compared to young to show anxiety and depression after ATR inhalation.

### 2.2. Effects of ATR Inhalation on Spatial Learning and Memory Function

**Figure 2:**
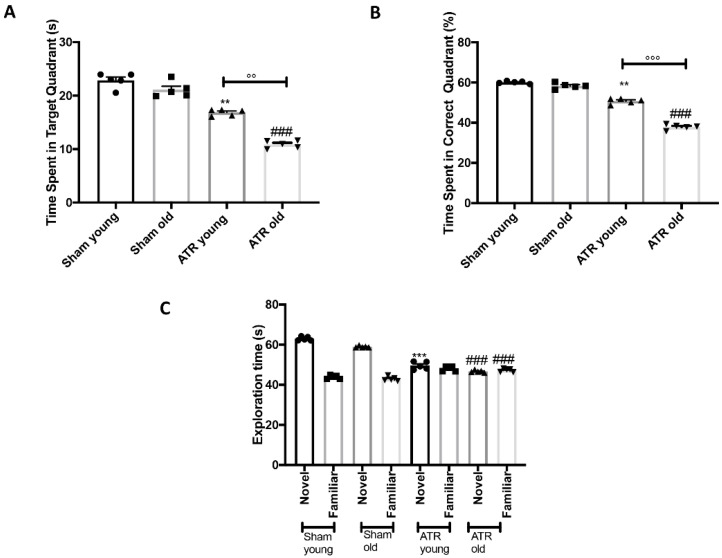
Effect of ATR inhalation on spatial learning and memory function. Morris Water Maze Test (**A**,**B**); Novel object recognition (**C**). MWM test showed that aged mice were more susceptible compared to young to develop spatial and memory deficits after ATR inhalation. No significant difference were found between young and aged mice during NOR. Data is expressed as the mean ± SEM of *n* = 5 animals for each group. ** *p* < 0.01 vs. Sham young; *** *p* < 0.001 vs. Sham young; ### *p* < 0.001 vs. Sham old; °° *p* < 0.01 ATR old vs. ATR young; °°° *p* < 0.001 ATR old vs. ATR young.

To determine the impact of ATR exposure on memory and learning the Morris Water Maze test was executed. The results revealed that, both young and aged mice exposed to ATR, showed spatial learning and memory deficits as demonstrated by the reduction in the time spent in target quadrant following ATR inhalation (Figure 2A,B). Also, in this case we observed that, when we compared the data of aged mice to young mice, the condition where considerably worsen. Moreover, in order to investigate impairments in their social interaction, we performed the Novel Object Recognition test. During this analysis we found that both young and aged mice were similarly affected and spend less time in the exploration of the novel or familiar objects compared to their controls, but we didn’t observe any statistically different across young or aged mice exposed to ATR (Figure 2C).

### 2.3. Changes in Motor Activity after ATR Inhalation

**Figure 3:**
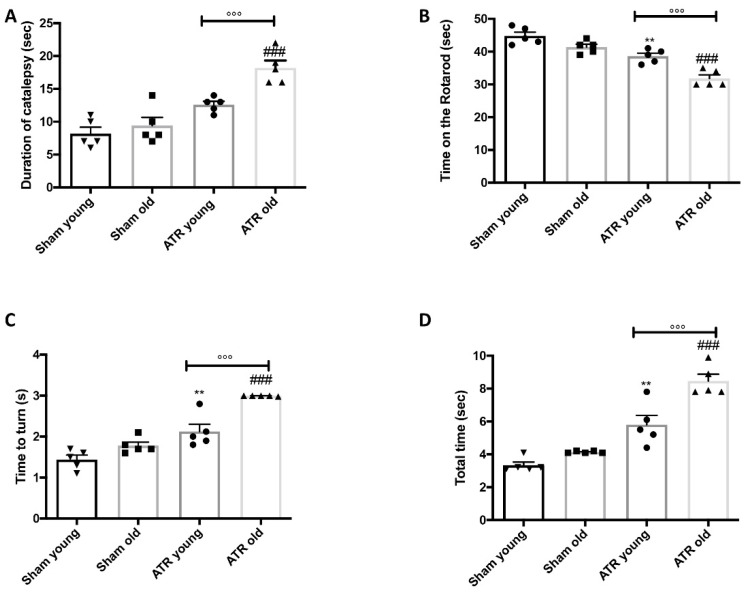
Effect of ATR inhalation on motor functions. Catalepsy test (**A**); Rotarod test (**B**); Time to turn (**C**) and Total time (**D**) for pole test. Aged mice were more susceptibility compared to young to develop motor deficits after ATR inhalation. Data is expressed as the mean ± SEM of *n* = 5 animals for each group. ** *p* < 0.01 vs. Sham young; ### *p* < 0.001 vs. Sham old; °°° *p* < 0.001 ATR old vs. ATR young.

It’s well known that ATR exposure causes dopaminergic alteration, but less is known about behavioral alteration dopaminergic-related [39,40,41,42,43,44,45]. Catalepsy test was used to investigate the effect of ATR inhalation on both young and aged mice (Figure 3A). After 28 days, both groups exhibited a significant increase of cataleptic symptoms compared to their controls. Additionally, through the Rotarod test, we also evaluated the motor function. At the end of experiments, significant motor changes were evidenced by the reduction in the time spent on the Rotarod (Figure 3B). Also, the pole test was used to assess whether the ATR inhalation induced bradykinesia [46]. “Time to turn” (Figure 3C) and “Total time” (Figure 3D) notably increased following ATR inhalation compared to the respectively sham. It is particularly important to note that not only in elderly mice was there a significant response, but that when analyzing all data it emerged that in elderly mice the response was considerably greater than in young mice.

### 2.4. Changes in Grip Strength and Sociability after ATR Exposure

**Figure 4:**
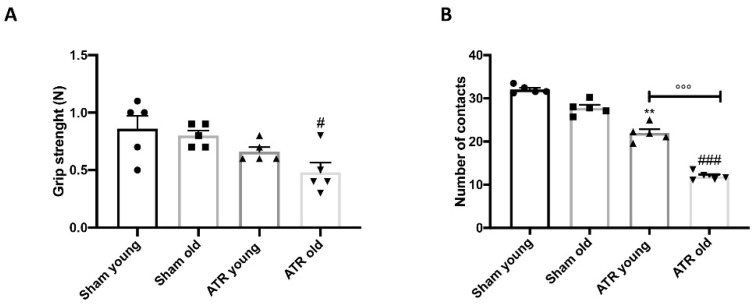
Effect of ATR inhalation on grip strength and sociability. Grip strength (**A**); Social interaction test (**B**). Aged mice were more susceptibility to develop a reduction in grip strength and social interaction compared to young mice after ATR inhalation. Data is expressed as the mean ± SEM of *n* = 5 animals for each group. ** *p* < 0.01 vs. Sham young; # *p* < 0.05 vs. Sham old; ### *p* < 0.001 vs. Sham old; °°° *p* < 0.001 ATR old vs. ATR young.

By the grip strength test (Figure 4A), we evaluated if ATR exposure altered mice muscle force. We found that after ATR inhalation, only aging mice showed a significant reduction in grip strength. No significantly difference were found between young and aged mice. Additionally, we found by the social interactions test (Figure 4B) that after ATR inhalation both groups decreased the number of contacts, indicating a deficit in social behavior. Interesting, we found that aged mice are more susceptible to develop this deficit compared to young group after ATR inhalation.

### 2.5. ATR Exposure Increases Oxidative Stress and Lipid Peroxidation

**Figure 5:**
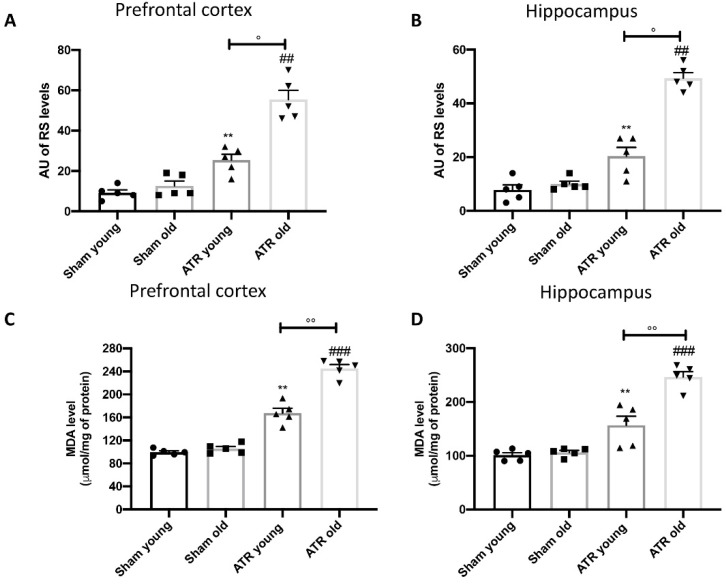
Effect of ATR inhalation on oxidative stress and lipid peroxidation. RS levels in prefrontal cortex (**A**); RS levels in hippocampus (**B**); MDA levels in prefrontal cortex (**C**); MDA levels in hippocampus (**D**). After ATR exposure, both young and aged mice showed a significant increase in RS production as well as in lipid peroxidation in prefrontal cortex and hippocampus. Statistical analysis reveal that aged mice were more susceptibility compared to young mice to develop RS and lipid peroxidation. Data is expressed as the mean ± SEM of *n* = 5 animals for each group. ** *p* < 0.01 vs. Sham young; ## *p* < 0.01 vs. Sham old; ### *p* < 0.001 vs. Sham old; ° *p* < 0.05 ATR old vs. ATR young; °° *p* < 0.01 ATR old vs. ATR young.

It’s well known that oxidative stress generated by reactive species (RS) is directly implicated in cognitive impairment and several neuropathological manifestations of aging. For this reason, we evaluated RS levels in both prefrontal cortex (Figure 5A) and hippocampus (Figure 5B). We found that after ATR exposure both young and aged mice demonstrated a significantly increase in RS levels in prefrontal cortex as well as in hippocampus, compared with their respectively controls. In particular, statistical analysis revealed that aged mice are more subject to develop RS compared to young mice. Additionally, by malondialdehyde (MDA) evaluation, we found a significantly increased lipid peroxidation in prefrontal cortex (Figure 5C) as well as in hippocampus (Figure 5D) in both groups. Also, in this case we found that after ATR exposure, aged mice are more susceptibility compared to young mouse.

### 2.6. ATR Inhalation Causes an Imbalance in Physiological Antioxidant Response

**Figure 6:**
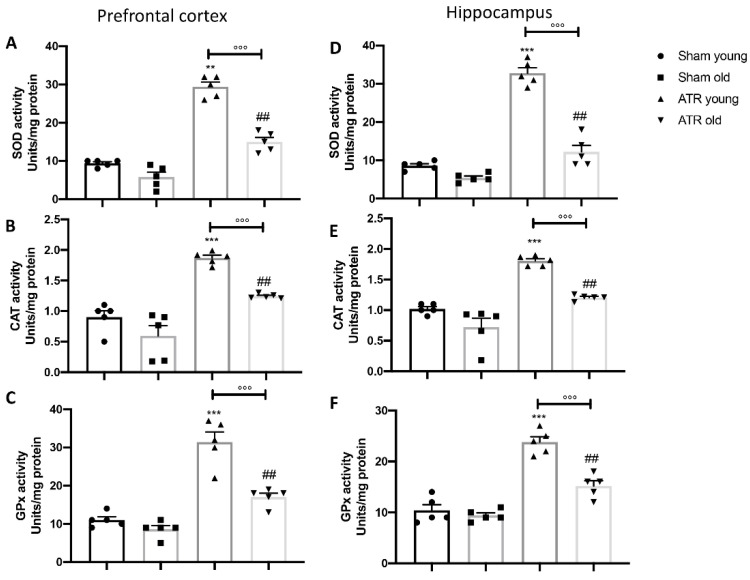
Effect of ATR inhalation on SOD, CAT and GPx activity. By ELISA kit we investigated the effect of ATR inhalation on SOD, CAT and GPx activity in prefrontal cortex (**A–C**) as well as in the hippocampus (**D–F**). After ATR exposure, both young and aged mice showed a significant increase in physiological antioxidant response in prefrontal cortex and hippocampus. Statistical analysis revealed that older mice showed less physiological response than younger mice. Data is expressed as the mean ± SEM of *n* = 5 animals for each group. ** *p* < 0.01 vs. Sham young; *** *p* < 0.001 vs. Sham young; ## *p* < 0.01 vs. Sham old; °°° *p* < 0.001 ATR old vs. ATR young.

In order to further explore the effect of ATR inhalation on the antioxidant response of cells, by ELISA kit, we investigated the activity of superoxide dismutase (SOD) (Figure 6A,D) as well as of catalase (CAT) (Figure 6B,E) and glutathione peroxidase (GPx) (Figure 6C,F). We found that in both prefrontal cortex and hippocampus, ATR exposure stimulated the physiological antioxidant response compared to their respective control, but in aged mice this response was less compared to young group exposed to ATR.

### 2.7. ATR Inhalation Induces Brain Apoptosis

**Figure 7:**
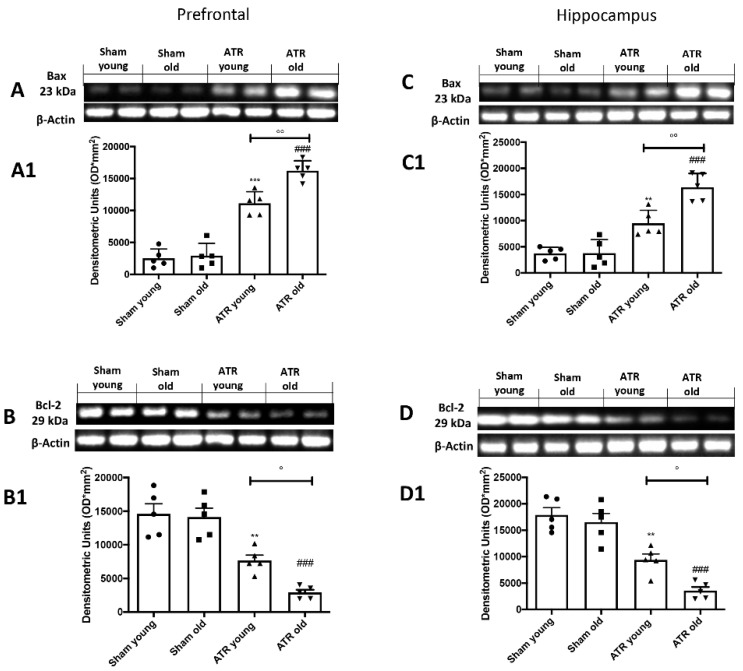
Impact of ATR inhalation on brain apoptosis. Western blots and respectively quantification of Bax in prefrontal cortex (**A**,**A1**) and hippocampus (**C**,**C1**) and Bcl-2 in prefrontal cortex (**B**,**B1**) and hippocampus (**D**,**D1**) We found that those aged mice are more subjected to brain apoptosis compared to young mice after 28 days of ATR inhalation. Data is expressed as the mean ± SEM of *n* = 5 animals for each group. ** *p* < 0.01 vs. Sham young; *** *p* < 0.01 vs. Sham young; ### *p* < 0.001 vs. Sham old; ° *p* < 0.05 ATR old vs. ATR young; °° *p* < 0.01 ATR old vs. ATR young.

By western blots, we investigated the impact of ATR inhalation on brain apoptosis. We found that ATR inhalation after 28 days, induces a significantly increase in Bax expression in both groups in prefrontal cortex (Figure 7A, see densitometric analysis A1) as well as in the hippocampus (Figure 7C, see densitometric analysis C1). On the other hand, we found a significantly decrease in Bcl-2 in both groups in prefrontal cortex (Figure 7B, see densitometric analysis B1) as well as in the hippocampus (Figure 7D, see densitometric analysis D1). Our analysis highlights those ATR-treated old mice are more subjected to brain apoptosis compared to young mice.

### 2.8. ATR Inhalation Induces Brain Inflammation

**Figure 8:**
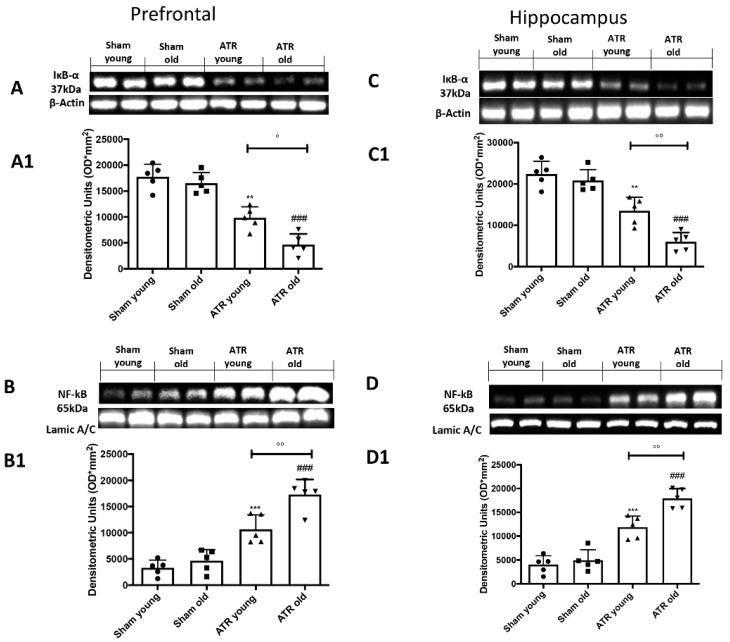
Impact of ATR inhalation on brain inflammation. Western blots and respectively quantification of ik-bα in prefrontal cortex (**A**,**A1**) and hippocampus (**C**,**C1**) and nf-kb in prefrontal cortex (**B**,**B1**) and hippocampus (**D**,**D1**). We found that aged mice are more subjected to brain inflammation compared to young mice after 28 days of ATR inhalation. Data is expressed as the mean ± SEM of *n* = 5 animals for each group. ** *p* < 0.01 vs. Sham young; *** *p* < 0.001 vs. Sham young; ### *p* < 0.001 vs. Sham old; ° *p* < 0.05 ATR old vs. ATR young; °° *p* < 0.01 ATR old vs. ATR young.

By western blots, we investigated if ATR inhalation could induce brain inflammation. We found that after ATR inhalation, in both young and aged mice we found a significant decrease in Iк-bα expression in both groups in the prefrontal cortex (Figure 8A, see densitometric analysis A1) as well as in the hippocampus (Figure 8C, see densitometric analysis C1). On the other hand, we found a significantly increase in nf-kb in both groups in the prefrontal cortex (Figure 8B, see densitometric analysis B1) as well as in the hippocampus (Figure 8D, see densitometric analysis D1). Our analysis highlights those aged mice are more subjected to brain inflammation compared to young mice.

### 2.9. ATR Inhalation Induces Pro-Inflammatory Cytokines Release

**Figure 9:**
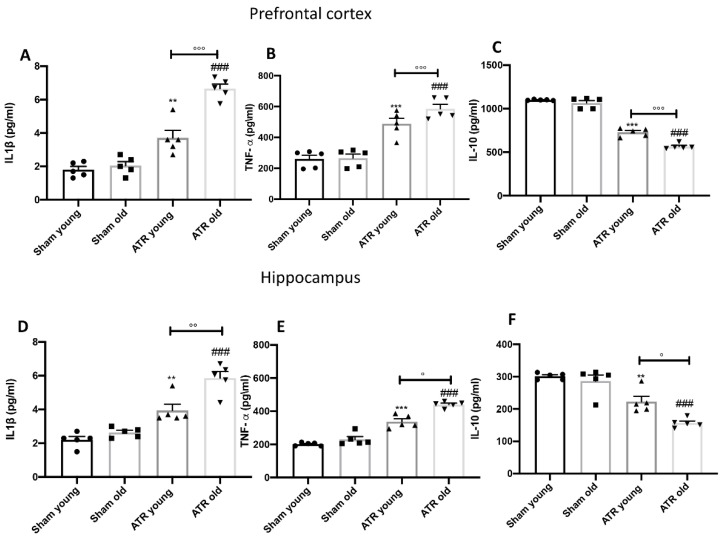
Impact of ATR inhalation on cytokines production. ELISA kit of IL-1β (**A**) and TNF-α (**B**) and IL-10 (**C**) in prefrontal cortex. ELISA kit of IL-1β (**D**) and TNF-α (**E**) and IL-10 (**F**) in hippocampus. After ATR exposure, both young and aged mice showed a significantly increase in pro-inflammatory cytokines production and a significantly decrease in IL-10 release in prefrontal cortex as well as in hippocampus. Statistical analysis reveal that older mice showed a major inflammatory response than younger mice. Data is expressed as the mean ± SEM of *n* = 5 animals for each group. ** *p* < 0.01 vs. Sham young; *** *p* < 0.001 vs. Sham young; ### *p* < 0.001 vs. Sham old; ° *p* < 0.05 ATR old vs. ATR young. °° *p* < 0.01 ATR old vs. ATR young; °°° *p* < 0.001 ATR old vs. ATR young.

The transcription factor nf-κb regulates a lot of mediators of inflammatory responses, including cytokines. By ELISA Kit, we found that 28 days after ATR inhalations, in both prefrontal cortex (Figure 9A for interleukin-1β (IL-1β) and B for tumor necrosis factor-α (TNF-α)) and hippocampus (Figure 9D for IL-1β and E for TNF-α), of young mice as well as in aged mice, there was a significant increase in pro-inflammatory cytokine release. On the other hand, we found a significant decrease in IL-10 production (Figure 9C for prefrontal cortex and F for hippocampus). Interestingly, aged mice showed a major inflammatory response and a minor anti-inflammatory response when exposed to ATR compared to the young group.

## 3. Discussion

Exposure to low amounts of numerous substances over a lifetime may be important in influencing the rate at which the brain ages [47]. This is a challenging problem to solve because of the intricacy of the exogenous substance that people are exposed to everyday. It is mandatory to make a variety of behavioral and biological analyses, as well as an examination of the differences in the responses elicited by younger and older persons exposed to these molecules to improve the knowledge of the brain alterations that occurs after exposure.

ATR is a commonly used herbicide for controlling broadleaf weeds. It is a man-made compound that does not exist naturally and is widely used on corn crops in the United States and Europe. The United States Environmental Protection Agency (EPA) has designated ATR as a restricted use pesticide (RUP), meaning that only licensed herbicide users can purchase or use it due to its persistence in water and various adverse health effects on humans [48]. Unlike the United States, EU has stricter regulations on the use of ATR. A pesticide directive issued by the EU, in 1991, restricted the use of chemicals that were accused of causing harm to human health, groundwater, or the atmosphere. As a result of this discovery, a regulatory ban on ATR was enacted in 2005, affecting all EU member states [49]. Significant quantities of ATR that are not absorbed by plants do end up in the environment. ATR is only weakly adsorbed by soil particles after application, and thus mainly leaves the field in runoff water. Rainfall washes large quantities of ATR out of the soil and into nearby areas, such as streams, reservoirs, and other waterways. Moreover, after it is added to the soil, small quantities of ATR may reach the air [50]. Humans are mainly exposed to ATR by the intake of tainted drinking water. However, inhalation exposure may occur during application. ATR’s negative effects are still being studied [51]. In humans, increased risk of intrauterine growth retardation, decreased semen content, and spontaneous abortions were found in many peer-reviewed studies, as were demasculinization and hermaphrodism in frogs [52,53,54,55,56]. Different studies have been recently demonstrated that exposure to ATR induce a dopaminergic and serotonergic toxicity, an alteration in GABA, glutamate and glutamine markers and a lot of behavioral alteration [32,45,57,58,59,60,61,62].

All these studies have in common the single or repeated oral or IP administration for short or long period. But until today, nobody investigated the impact of ATR inhalation on brain, in particular during different life time.

In our study, after 28 days of daily exposure to an aerosol containing 25 mg/kg of ATR, we found that in both young and aged mice there was an increase in anxiety and depression and a deficit in spatial learning and memory function as well as an alteration in motor activity, grip strength and sociability. In particular, excluding the social interaction and the grip strength we found that the alteration was more marked in aged mice compared to young mice.

With this idea in our mind, we investigated the effect of ATR inhalation on oxidative stress and lipid peroxidation, and we found a significant increase in both young and aged mice in the prefrontal cortex as well as in the hippocampus with an intensification in aged mice more that young mice. As it is well known, in order to respond to ROS and RNS formation, cells activated SOD, CAT, and GPx system. We found that this response was significantly compromised in aged mice subjected to ATR inhalations.

Excess cellular levels of ROS cause damage to proteins, nucleic acids, lipids, membranes and organelles, which can start to nf-kb pathway activation and at last lead to apoptosis [63]. As we supposed, we found a significant increase in this deleterious pathway, as well as to a pro inflammatory cytokine production, in both hippocampus and prefrontal cortex in young as well as aged mice. Also, in this investigation the response in aged mice were more intense compared to young mice.

This research aims to investigate the effects of ATR exposure on both and aged mice, with particular attention to the difference in the physiological response put in place by the cells during aging. We demonstrate that after ATR exposure, aged mice are more susceptible compared to young mice to develop behavioral alterations, in particular in anxiety, depression, spatial learning and memory function, as well as in motor impairment and sociability. Additionally, in aged mice, after ATR exposure we found a significant increase in oxidative stress and apoptosis with a reduced response of physiological antioxidant system. This is the first study that considers ATR as an air contaminant that can compromise physiological aging by accelerating and perturbating the entire process. In the future it would be interesting to investigate the systemic effect of ATR inhalation on different organs, in different health conditions, with particular attention to dementia and pulmonary fibrosis.

## 4. Materials and Methods

### 4.1. Animals

Young and old CD1 mice (male, 8 week old, 18–24 g and 24 month old, 25–30 g) were acquired from Envigo (Milan, Italy) and posted in a controlled environment [64]. The study was approved by the Review Board of the University of Messina for the care of animals (266/2021-PR). All animal experiments were in compliance with the new Italian regulations (D.Lgs 2014/26), the EU regulations (EU Directive 2010/63) and the ARRIVE guidelines.

### 4.2. Experimental Design and Groups

The ATR aerosol was prepared by dissolving 25 mg of ATR (Merck, Darmstadt, Germany) in a vehicle made with saline and 10% DMSO. After complete solubilization, a Lovelace nebulizer (In-Tox Products, Albuquerque, NM, USA) was used to create an atmosphere in an exposure chamber (Research and Consulting Co., AG, Basel, Switzerland) [65,66,67]. In detail, each mouse (six for group) was carefully inserted into an animal tube with the nose pointing to the aerosol outlet. The animal tubes were specifically designed to contain one mouse per tube. Using the plunger in the tube, the mouse was gently immobilized in the correct position. This phase was very important to allow the animal to breathe properly. After being immobilized, a known volume of vehicle or ATR (pro kilo) was placed in the nebulization until it was completely nebulized.

The mice were randomly divided into the following two groups:

(I) Sham group, i.e., animals that were exposed to the vehicle (saline with 10% of DMSO).
(II) ATR group, i.e., animals that were exposed to 25 mg of ATR every day for 28 days.

After exposure, the mice were housed in individual cages and maintained under a 12:12 h light/dark cycle at 21  ±  1 °C and 50  ±  5% humidity. Standard laboratory litter, diet, and water were available ad libitum. Additionally, the mice were weighted and observed for any clinical symptoms, and the information was recorded by the animal care staff. At the end of experiment, the mice were sacrificed by cervical dislocation under anesthesia and brain were collected for different analysis, as previously described [37,38,65,68,69,70]. The ATR dosage was chosen based on other previous studies, but for the first time, ATR was not administered by oral gavage but instead by aerosol, because there is still limited knowledge of the effects of ATR on the brain [45,58,71] (Figure S1).

### 4.3. Behavioral Testing

In another set of experiments, the same group previously described was subjected to behavioral tests at 1 and 28 days of experiment. Mice were transferred to the behavior testing room 30 min prior to beginning the first trial to habituate to the condition of the behavior testing room. Animals were familiarized to the apparatus before every recording based on behavioral test which were subjected to keep the condition as uniform as possible.

Three different reliable expert observers blinded to the injury status of the animals conducted and analyzed the behavioral tests. Tests are described below:

#### 4.3.1. Pole Test (PT)

A pole test (PT) was performed to detect motor alteration, as previously described [72,73]. Briefly, mice after a training were positioned with their head oriented upward on top of the pole and time to T turn and total time to descend was recorded for five different trials.

#### 4.3.2. Rotarod Test (RT)

Motor activity was assessed with a rotary rod apparatus using a protocol previously described [74,75]. Concisely, after habituation animal was placed back on the drum of instrument immediately after falling, up to 5 times in one session.

#### 4.3.3. Catalepsy Test (CT)

Catalepsy, was measured as previously described [76,77]. In particular, mice were positioned so that their hindquarters were on the bench. The length of time the mice maintained this position was recorded.

#### 4.3.4. Elevated Plus-Maze (EPM)

The Elevated pluz-maze Test (EPMT) test was performed to evaluate the anxiety state as described previously [78]. After a training the number of times mice went into each arm and the time in open arms were recorded.

#### 4.3.5. Open Field Test (OFT)

Locomotor activity and anxiety-like behavior were monitored for 5 min using the OFT. After a training, each mouse was placed in the center of the box and activity was scored [79].

#### 4.3.6. Morris Water Maze (MWM)

MWM test was used to evaluate hippocampal-dependent spatial learning and memory function [80,81]. After a training, mouse was located into the water in each of the three different quadrants and allowed to swim for 1 min each time. One day after the navigation experiment, the platform was removed for the test. The time spent in the target quadrant was recorded.

#### 4.3.7. Grip Strength Test

Briefly, mice were carefully placed in front of the wire grid and allowed to grab hold with both fore paws. Once grip was established, the maximum grip strength was recorded (in Newtons). For each animal, 4 measurements (1 min apart) were taken to obtain an average value.

#### 4.3.8. Forced Swim Test (FST)

The technique is based on that explained by Porsolt et al. [82]. Each mouse was softly placed in the cylinder for 6 min, and the length of floating was scored. Immobility was analyzed during the last 4-min period of the test.

#### 4.3.9. Novel Object Recognition (NOR) Test

The spontaneous inclination of mice to spend time investigating a novel object or a familiar one was examined with the NOR test. After a training period, mice were placed in the box for a 5 min session and the examinator randomly exchanged one of the familiar objects with a novel one. The total time the mouse spent exploring each object was recorded [83,84].

#### 4.3.10. Social Interaction Test

The social interaction test consisted of three trials of ten minutes. Initially, a mouse was acclimated in an empty arena. In the second phase, the experimental mouse was exposed to an object. In the third phase the experimental mouse was exposed to an object in which it was placed with other animals [85,86].

### 4.4. Western Blot Analysis of Cytosolic and Nuclear Extracts

Extracts of the cytosol and nucleus were prepared, as previously mentioned [84,87,88,89,90]. The following primary antibodies were used: anti-Bax (1:500, Santa Cruz Biotechnology, #sc7480), anti-Bcl-2 (1:500, Santa Cruz Biotechnology, #sc7382), anti-Iκbα (1:500, Santa Cruz Biotechnology, #sc-1643), and anti-nfκb (1:500, Santa Cruz Biotechnology, #sc8414) in 1× PBS, 5% *w/v* non-fat dried milk, and 0.1% Tween 20, at 4 °C overnight [91,92,93,94]. For the cytosolic fraction, Western blots were also probed with antibody against β-actin protein (1:500, Santa Cruz Biotechnology, Dallas, TX, USA). The same methods were used for nuclear fraction with lamin A/C (1:500, Sigma-Aldrich Corp., Milan, Italy) [95,96]. Signals were examined with an enhanced chemiluminescence (ECL) detection system reagent, according to the manufacturer’s instructions (Thermo, Monza, Italy). The relative expression of the protein bands was quantified by densitometry with BIORAD ChemiDocTM XRS+ software [84,91,97,98,99].

### 4.5. Evaluation of Tissue Lipid Peroxidation

Malonaldehyde (MDA) levels was assessed, as previously described for brain tissue, at the end of the experiments. Briefly, after homogenization with opportune buffer, MDA absorbances was measured at 650 nm, using a spectrophotometer and expressed in mill-units per 100 milligram weights (mU/100 mg) of wet tissue [96,100,101,102,103,104,105].

### 4.6. Cytokine Measurement

The hippocampus and prefrontal cortex were dissected from half of the whole brains. Briefly, the supernatant of homogenate of both brain tissue was centrifuged and were measured using ELISA kits (R&D Systems, Minneapolis, MN, USA) following the manufacturer’s instructions. The absorbance value of each well was measured at 450 nm by a microplate reader [97,98,106,107,108,109,110].

### 4.7. Reactive Species (RS) Detemination

To estimate the RS production in the prefrontal cortex and hippocampus we used a consolidated method of DCHF-DA assay as previously described by Loetchutinat et al. [111]. DCHF-DA (Sigma-Aldrich Corp., Milan, Italy) is a nonfluorescent compound that easily crosses cell membranes and, in the presence of RS is rapidly oxidized to its highly fluorescent derivative dichlorofluorescein (DCF). The DCF fluorescence intensity emission was recorded at 520 nm and RS levels were expressed as arbitrary unit (AU).

### 4.8. SOD, CAT and GPx Evaluation

SOD activity was assayed spectrophotometrically according to the method described by Misra and Fridovich and the color reaction was measured at 480 nm and expressed as Units (U)/mg protein [112,113].

CAT activity was spectrophotometrically measured by the method proposed by Aebi [113]. The enzymatic activity was expressed as Units (U)/mg protein (1U decomposes 1 μmol H2O2/min at pH 7 at 25 °C).

GPx activity was assayed spectrophotometrically by the method of Wendel, through the glutathione (GSH)/NADPH/glutathione reductase system, by the dismutation of H_2_O_2_ at 340 nm [113,114]. The enzymatic activity was expressed in nmol NADPH/min/mg protein.

### 4.9. Materials

Unless otherwise stated, all compounds were purchased from Sigma-Aldrich.

### 4.10. Statistical Evaluation

In this study, the data are expressed as the average ± SEM and represent at least 3 experiments carried out in different days. For in vivo studies, N represents the number of animals used. The number of animals used for in vivo studies was carried out by G*Power 3.1 software (Die Heinrich-Heine-Universität Düsseldorf, Düsseldorf, Germany). Data were analyzed by an experienced histopathologist, and all the studies were performed without knowledge of the treatments. The results were analyzed by one-way ANOVA followed by a Bonferroni post-hoc test for multiple comparisons. A *p* value less than 0.05 was considered significant.

## Data Availability

The data used to support the findings of this study are available from the corresponding author upon request.

## Supplementary Materials

The following are available online at https://www.mdpi.com/article/10.3390/ijms22157938/s1, Figure S1: Experimental flow chart.

## References

[1] Bondy S.C. (2016). Anthropogenic pollutants may increase the incidence of neurodegenerative disease in an aging population. Toxicology.

[2] Sharman E.H., Bondy S.C., Sharman K.G., Lahiri D., Cotman C.W., Perreau V.M. (2007). Effects of melatonin and age on gene expression in mouse CNS using microarray analysis. Neurochem. Int..

[3] Bondy S.C., Sharman E.H. (2007). Melatonin and the aging brain. Neurochem. Int..

[4] Bondy S.C., Lahiri D.K., Perreau V.M., Sharman K.Z., Campbell A., Zhou J., Sharman E.H. (2004). Retardation of brain aging by chronic treatment with melatonin. Ann. N. Y. Acad. Sci..

[5] Sharman E.H., Vaziri N.D., Ni Z., Sharman K.G., Bondy S.C. (2002). Reversal of biochemical and behavioral parameters of brain aging by melatonin and acetyl L-carnitine. Brain Res..

[6] Qin L., Wu X., Block M.L., Liu Y., Breese G.R., Hong J.S., Knapp D.J., Crews F.T. (2007). Systemic LPS causes chronic neuroinflammation and progressive neurodegeneration. Glia.

[7] Becaria A., Lahiri D.K., Bondy S.C., Chen D., Hamadeh A., Li H., Taylor R., Campbell A. (2006). Aluminum and copper in drinking water enhance inflammatory or oxidative events specifically in the brain. J. Neuroimmunol..

[8] Cambier S., Gonzalez P., Mesmer-Dudons N., Brethes D., Fujimura M., Bourdineaud J.P. (2012). Effects of dietary methylmercury on the zebrafish brain: Histological, mitochondrial, and gene transcription analyses. Biometals.

[9] Sipos E., Chen L., Andras I.E., Wrobel J., Zhang B., Pu H., Park M., Eum S.Y., Toborek M. (2012). Proinflammatory adhesion molecules facilitate polychlorinated biphenyl-mediated enhancement of brain metastasis formation. Toxicol. Sci..

[10] Santos D., Batoreu M.C., Tavares de Almeida I., Davis Randall L., Mateus M.L., Andrade V., Ramos R., Torres E., Aschner M., Marreilha dos Santos A.P. (2013). Evaluation of neurobehavioral and neuroinflammatory end-points in the post-exposure period in rats sub-acutely exposed to manganese. Toxicology.

[11] Backman L., Nyberg L., Lindenberger U., Li S.C., Farde L. (2006). The correlative triad among aging, dopamine, and cognition: Current status and future prospects. Neurosci. Biobehav. Rev..

[12] Dalphin J.C. (1998). [Respiratory pathology in the agricultural environment]. Rev. Prat..

[13] Hoppin J.A., Umbach D.M., London S.J., Alavanja M.C., Sandler D.P. (2003). Animal production and wheeze in the Agricultural Health Study: Interactions with atopy, asthma, and smoking. Occup. Environ. Med..

[14] Song X.Y., Li J.N., Wu Y.P., Zhang B., Li B.X. (2015). Atrazine Causes Autophagy- and Apoptosis-Related Neurodegenerative Effects in Dopaminergic Neurons in the Rat Nigrostriatal Dopaminergic System. Int. J. Mol. Sci..

[15] Guzzella L., Pozzoni F., Giuliano G. (2006). Herbicide contamination of surficial groundwater in Northern Italy. Environ. Pollut..

[16] Abraxis L., Kit A.E., Kit A.T. (2007). Immunoassay Test. Kits for Atrazine.

[17] EPAUS (2003). Interim Reregistration Eligibility Decision for Atrazine.

[18] Singh M., Kaur P., Sandhir R., Kiran R. (2008). Protective effects of vitamin E against atrazine-induced genotoxicity in rats. Mutat Res..

[19] Simpkins J.W., Swenberg J.A., Weiss N., Brusick D., Eldridge J.C., Stevens J.T., Handa R.J., Hovey R.C., Plant T.M., Pastoor T.P. (2017). Atrazine and Breast Cancer: A Framework Assessment of the Toxicological and Epidemiological Evidence. Toxicol. Sci..

[20] Inoue-Choi M., Weyer P.J., Jones R.R., Booth B.J., Cantor K.P., Robien K., Ward M.H. (2016). Atrazine in public water supplies and risk of ovarian cancer among postmenopausal women in the Iowa Women’s Health Study. Occup. Environ. Med..

[21] Hu K., Tian Y., Du Y., Huang L., Chen J., Li N., Liu W., Liang Z., Zhao L. (2016). Atrazine promotes RM1 prostate cancer cell proliferation by activating STAT3 signaling. Int. J. Oncol..

[22] Albanito L., Lappano R., Madeo A., Chimento A., Prossnitz E.R., Cappello A.R., Dolce V., Abonante S., Pezzi V., Maggiolini M. (2015). Effects of atrazine on estrogen receptor alpha-and G protein-coupled receptor 30-mediated signaling and proliferation in cancer cells and cancer-associated fibroblasts. Environ. Health Perspect..

[23] Boffetta P., Adami H.O., Berry S.C., Mandel J.S. (2013). Atrazine and cancer: A review of the epidemiologic evidence. Eur. J. Cancer Prev..

[24] Freeman L.E., Rusiecki J.A., Hoppin J.A., Lubin J.H., Koutros S., Andreotti G., Zahm S.H., Hines C.J., Coble J.B., Barone-Adesi F. (2011). Atrazine and cancer incidence among pesticide applicators in the agricultural health study (1994-2007). Environ. Health Perspect..

[25] McElroy J.A., Gangnon R.E., Newcomb P.A., Kanarek M.S., Anderson H.A., Brook J.V., Trentham-Dietz A., Remington P.L. (2007). Risk of breast cancer for women living in rural areas from adult exposure to atrazine from well water in Wisconsin. J. Expo. Sci. Environ. Epidemiol..

[26] Rusiecki J.A., De Roos A., Lee W.J., Dosemeci M., Lubin J.H., Hoppin J.A., Blair A., Alavanja M.C. (2004). Cancer incidence among pesticide applicators exposed to atrazine in the Agricultural Health Study. J. Natl. Cancer Inst..

[27] Hessel P.A., Kalmes R., Smith T.J., Lau E., Mink P.J., Mandel J. (2004). A nested case-control study of prostate cancer and atrazine exposure. J. Occup. Environ. Med..

[28] Van Leeuwen J.A., Waltner-Toews D., Abernathy T., Smit B., Shoukri M. (1999). Associations between stomach cancer incidence and drinking water contamination with atrazine and nitrate in Ontario (Canada) agroecosystems, 1987–1991. Int. J. Epidemiol..

[29] Xing H., Li S., Wang Z., Gao X., Xu S., Wang X. (2012). Histopathological changes and antioxidant response in brain and kidney of common carp exposed to atrazine and chlorpyrifos. Chemosphere.

[30] Schmidel A.J., Assmann K.L., Werlang C.C., Bertoncello K.T., Francescon F., Rambo C.L., Beltrame G.M., Calegari D., Batista C.B., Blaser R.E. (2014). Subchronic atrazine exposure changes defensive behaviour profile and disrupts brain acetylcholinesterase activity of zebrafish. Neurotoxicol. Teratol..

[31] Horzmann K.A., Reidenbach L.S., Thanki D.H., Winchester A.E., Qualizza B.A., Ryan G.A., Egan K.E., Hedrick V.E., Sobreira T.J.P., Peterson S.M. (2018). Embryonic atrazine exposure elicits proteomic, behavioral, and brain abnormalities with developmental time specific gene expression signatures. J. Proteom..

[32] Chavez-Pichardo M.E., Reyes-Bravo D.Y., Mendoza-Trejo M.S., Marin-Lopez A.G., Giordano M., Hernandez-Chan N., Dominguez-Marchan K., Ortega-Rosales L.C., Rodriguez V.M. (2020). Brain alterations in GABA, glutamate and glutamine markers after chronic atrazine exposure in the male albino rat. Arch. Toxicol..

[33] Abarikwu S.O. (2014). Protective effect of quercetin on atrazine-induced oxidative stress in the liver, kidney, brain, and heart of adult wistar rats. Toxicol. Int..

[34] Mizota K., Ueda H. (2006). Endocrine disrupting chemical atrazine causes degranulation through Gq/11 protein-coupled neurosteroid receptor in mast cells. Toxicol. Sci..

[35] Jestadi D.B., Phaniendra A., Babji U., Srinu T., Shanmuganathan B., Periyasamy L. (2014). Effects of short term exposure of atrazine on the liver and kidney of normal and diabetic rats. J. Toxicol..

[36] Zhao F., Li K., Zhao L., Liu J., Suo Q., Zhao J., Wang H., Zhao S. (2014). Effect of Nrf2 on rat ovarian tissues against atrazine-induced anti-oxidative response. Int. J. Clin. Exp. Pathol..

[37] Zhang X., Wang M., Gao S., Ren R., Zheng J., Zhang Y. (2011). Atrazine-induced apoptosis of splenocytes in BALB/C mice. BMC Med..

[38] Liu W., Du Y., Liu J., Wang H., Sun D., Liang D., Zhao L., Shang J. (2014). Effects of atrazine on the oxidative damage of kidney in Wister rats. Int. J. Clin. Exp. Med..

[39] Li P., Li X., Yao L., Wu Y., Li B. (2020). Soybean isoflavones prevent atrazine-induced neurodegenerative damage by inducing autophagy. Ecotoxicol. Environ. Saf..

[40] Figueira F.H., de Quadros Oliveira N., de Aguiar L.M., Escarrone A.L., Primel E.G., Barros D.M., da Rosa C.E. (2017). Exposure to atrazine alters behaviour and disrupts the dopaminergic system in Drosophila melanogaster. Comp. Biochem. Physiol. Toxicol. Pharmacol..

[41] Li Y.S., He X., Ma K., Wu Y.P., Li B.X. (2015). The Effect of Exposure to Atrazine on Dopaminergic Development in Pubertal Male SD Rats. Birth. Defects Res. Dev. Reprod. Toxicol..

[42] Sun Y., Li Y.S., Yang J.W., Yu J., Wu Y.P., Li B.X. (2014). Exposure to atrazine during gestation and lactation periods: Toxicity effects on dopaminergic neurons in offspring by downregulation of Nurr1 and VMAT2. Int. J. Mol. Sci..

[43] Lin Z., Dodd C.A., Filipov N.M. (2013). Differentiation state-dependent effects of in vitro exposure to atrazine or its metabolite diaminochlorotriazine in a dopaminergic cell line. Life Sci..

[44] Bardullas U., Giordano M., Rodriguez V.M. (2011). Chronic atrazine exposure causes disruption of the spontaneous locomotor activity and alters the striatal dopaminergic system of the male Sprague-Dawley rat. Neurotoxicol Teratol..

[45] Coban A., Filipov N.M. (2007). Dopaminergic toxicity associated with oral exposure to the herbicide atrazine in juvenile male C57BL/6 mice. J. Neurochem..

[46] Ogawa N., Hirose Y., Ohara S., Ono T., Watanabe Y. (1985). A simple quantitative bradykinesia test in MPTP-treated mice. Res. Commun. Chem. Pathol. Pharm..

[47] Lucchini R., Zimmerman N. (2009). Lifetime cumulative exposure as a threat for neurodegeneration: Need for prevention strategies on a global scale. Neurotoxicology.

[48] (EPA), U.S.E.P.A (2008). Atrazine Background.

[49] Ackerman F. (2007). The economics of atrazine. Int. J. Occup. Environ. Health.

[50] Pohl H., Kolman J. (2006). Interaction Profile for Atrazine, Deethylatrazine, Diazinon, Nitrate and Simazine.

[51] Kavlock R. (1999). Overview of endocrine disruptor research activity in the United States. Chemosphere.

[52] Munger R., Isacson P., Hu S., Burns T., Hanson J., Lynch C.F., Cherryholmes K., Van Dorpe P., Hausler W.J. (1997). Intrauterine growth retardation in Iowa communities with herbicide-contaminated drinking water supplies. Environ. Health Perspect..

[53] Arbuckle T.E., Lin Z., Mery L.S. (2001). An exploratory analysis of the effect of pesticide exposure on the risk of spontaneous abortion in an Ontario farm population. Environ. Health Perspect..

[54] Hayes T.B., Collins A., Lee M., Mendoza M., Noriega N., Stuart A.A., Vonk A. (2002). Hermaphroditic, demasculinized frogs after exposure to the herbicide atrazine at low ecologically relevant doses. Proc. Natl. Acad. Sci. USA.

[55] Swan S.H. (2003). Semen quality in relation to pesticide exposure in Missouri males. Mo Med..

[56] Swan S.H., Kruse R.L., Liu F., Barr D.B., Drobnis E.Z., Redmon J.B., Wang C., Brazil C., Overstreet J.W., Study for Future Families Research Group (2003). Semen quality in relation to biomarkers of pesticide exposure. Environ. Health Perspect..

[57] Rodriguez V.M., Limon-Pacheco J.H., Mendoza-Trejo M.S., Gonzalez-Gallardo A., Hernandez-Plata I., Giordano M. (2013). Repeated exposure to the herbicide atrazine alters locomotor activity and the nigrostriatal dopaminergic system of the albino rat. Neurotoxicology.

[58] Lin Z., Dodd C.A., Filipov N.M. (2013). Short-term atrazine exposure causes behavioral deficits and disrupts monoaminergic systems in male C57BL/6 mice. Neurotoxicol. Teratol..

[59] Li Y., Sun Y., Yang J., Wu Y., Yu J., Li B. (2014). The long-term effects of the herbicide atrazine on the dopaminergic system following exposure during pubertal development. Mutat. Res. Genet. Toxicol. Environ. Mutagen..

[60] Li Y., Sun Y., Yang J., Wu Y., Yu J., Li B. (2014). Age-dependent dopaminergic dysfunction following fetal exposure to atrazine in SD rats. Environ. Toxicol. Pharmacol..

[61] Lin Z., Roede J.R., He C., Jones D.P., Filipov N.M. (2014). Short-term oral atrazine exposure alters the plasma metabolome of male C57BL/6 mice and disrupts alpha-linolenate, tryptophan, tyrosine and other major metabolic pathways. Toxicology.

[62] Belloni V., Dessi-Fulgheri F., Zaccaroni M., Di Consiglio E., De Angelis G., Testai E., Santochirico M., Alleva E., Santucci D. (2011). Early exposure to low doses of atrazine affects behavior in juvenile and adult CD1 mice. Toxicology.

[63] Redza-Dutordoir M., Averill-Bates D.A. (2016). Activation of apoptosis signalling pathways by reactive oxygen species. Biochim. Biophys. Acta..

[64] Cordaro M., D’Amico R., Morabito R., Fusco R., Siracusa R., Peritore A.F., Impellizzeri D., Genovese T., Crupi R., Gugliandolo E. (2021). Physiological and Biochemical Changes in NRF2 Pathway in Aged Animals Subjected to Brain Injury. Cell. Physiol. Biochem..

[65] Schramm C.M., Puddington L., Wu C., Guernsey L., Gharaee-Kermani M., Phan S.H., Thrall R.S. (2004). Chronic inhaled ovalbumin exposure induces antigen-dependent but not antigen-specific inhalational tolerance in a murine model of allergic airway disease. Am. J. Pathol..

[66] Vaughan R.P., Szewczyk M.T., Lanosa M.J., Desesa C.R., Gianutsos G., Morris J.B. (2006). Adenosine sensory transduction pathways contribute to activation of the sensory irritation response to inspired irritant vapors. Toxicol. Sci..

[67] Ramona D., Monaco F., Fusco R., Peritore A.F., Genovese T., Impellizzeri D., Crupi R., Interdonato L., Sforza A.M., Gugliandolo E. (2021). Exposure to Atrazine Induces Lung Inflammation through Nrf2-HO1 and Beclin 1/LC3 Pathways. Cell. Physiol. Biochem..

[68] Lin J., Xia J., Zhao H.S., Hou R., Talukder M., Yu L., Guo J.Y., Li J.L. (2018). Lycopene Triggers Nrf2-AMPK Cross Talk to Alleviate Atrazine-Induced Nephrotoxicity in Mice. J. Agric. Food Chem..

[69] Li J., Li X., Bi H., Ma K., Li B. (2018). Developmental Exposure to Atrazine Impairs Spatial Memory and Downregulates the Hippocampal D1 Dopamine Receptor and cAMP-Dependent Signaling Pathway in Rats. Int. J. Mol. Sci..

[70] Gao S., Wang Z., Zhang C., Jia L., Zhang Y. (2016). Oral Exposure to Atrazine Induces Oxidative Stress and Calcium Homeostasis Disruption in Spleen of Mice. Oxid. Med. Cell. Longev..

[71] Filipov N.M., Pinchuk L.M., Boyd B.L., Crittenden P.L. (2005). Immunotoxic effects of short-term atrazine exposure in young male C57BL/6 mice. Toxicol. Sci..

[72] Sedelis M., Schwarting R.K., Huston J.P. (2001). Behavioral phenotyping of the MPTP mouse model of Parkinson’s disease. Behav. Brain Res..

[73] Cordaro M., Siracusa R., Crupi R., Impellizzeri D., Peritore A.F., D’Amico R., Gugliandolo E., Di Paola R., Cuzzocrea S. (2018). 2-Pentadecyl-2-Oxazoline Reduces Neuroinflammatory Environment in the MPTP Model of Parkinson Disease. Mol. Neurobiol..

[74] Fleming S.M., Mulligan C.K., Richter F., Mortazavi F., Lemesre V., Frias C., Zhu C., Stewart A., Gozes I., Morimoto B. (2011). A pilot trial of the microtubule-interacting peptide (NAP) in mice overexpressing alpha-synuclein shows improvement in motor function and reduction of alpha-synuclein inclusions. Mol. Cell. Neurosci..

[75] Siracusa R., Paterniti I., Cordaro M., Crupi R., Bruschetta G., Campolo M., Cuzzocrea S., Esposito E. (2018). Neuroprotective Effects of Temsirolimus in Animal Models of Parkinson’s Disease. Mol. Neurobiol..

[76] Araki T., Kumagai T., Tanaka K., Matsubara M., Kato H., Itoyama Y., Imai Y. (2001). Neuroprotective effect of riluzole in MPTP-treated mice. Brain Res..

[77] Paterniti I., Campolo M., Siracusa R., Cordaro M., Di Paola R., Calabrese V., Navarra M., Cuzzocrea S., Esposito E. (2017). Liver X receptors activation, through TO901317 binding, reduces neuroinflammation in Parkinson’s disease. PLoS ONE.

[78] Pellow S., Chopin P., File S.E., Briley M. (1985). Validation of open:closed arm entries in an elevated plus-maze as a measure of anxiety in the rat. J. Neurosci. Methods.

[79] Prut L., Belzung C. (2003). The open field as a paradigm to measure the effects of drugs on anxiety-like behaviors: A review. Eur. J. Pharmacol..

[80] Siebold L., Krueger A.C., Abdala J.A., Figueroa J.D., Bartnik-Olson B., Holshouser B., Wilson C.G., Ashwal S. (2020). Cosyntropin Attenuates Neuroinflammation in a Mouse Model of Traumatic Brain Injury. Front. Mol. Neurosci..

[81] Zhao P., Zhou R., Zhu X.Y., Liu G., Zhao Y.P., Ma P.S., Wu W., Niu Y., Sun T., Li Y.X. (2017). Neuroprotective Effects of Lycium barbarum Polysaccharide on Focal Cerebral Ischemic Injury in Mice. Neurochem. Res..

[82] Porsolt R.D., Bertin A., Blavet N., Deniel M., Jalfre M. (1979). Immobility induced by forced swimming in rats: Effects of agents which modify central catecholamine and serotonin activity. Eur. J. Pharm..

[83] Pan Z., Cui M., Dai G., Yuan T., Li Y., Ji T., Pan Y. (2018). Protective Effect of Anthocyanin on Neurovascular Unit in Cerebral Ischemia/Reperfusion Injury in Rats. Front. Neurosci..

[84] Siracusa R., Impellizzeri D., Cordaro M., Crupi R., Esposito E., Petrosino S., Cuzzocrea S. (2017). Anti-Inflammatory and Neuroprotective Effects of Co-UltraPEALut in a Mouse Model of Vascular Dementia. Front. Neurol..

[85] Srivastava P., Cronin C.G., Scranton V.L., Jacobson K.A., Liang B.T., Verma R. (2020). Neuroprotective and neuro-rehabilitative effects of acute purinergic receptor P2X4 (P2X4R) blockade after ischemic stroke. Exp. Neurol..

[86] Boccella S., Iannotta M., Cristiano C., Iannotti F.A., Bello F.D., Guida F., Belardo C., Infantino R., Ricciardi F., Giannella M. (2020). Treatment With 2-Pentadecyl-2-Oxazoline Restores Mild Traumatic Brain Injury-Induced Sensorial and Neuropsychiatric Dysfunctions. Front. Pharm..

[87] Cordaro M., Paterniti I., Siracusa R., Impellizzeri D., Esposito E., Cuzzocrea S. (2017). KU0063794, a Dual mTORC1 and mTORC2 Inhibitor, Reduces Neural Tissue Damage and Locomotor Impairment After Spinal Cord Injury in Mice. Mol. Neurobiol..

[88] Campolo M., Esposito E., Ahmad A., Di Paola R., Paterniti I., Cordaro M., Bruschetta G., Wallace J.L., Cuzzocrea S. (2014). Hydrogen sulfide-releasing cyclooxygenase inhibitor ATB-346 enhances motor function and reduces cortical lesion volume following traumatic brain injury in mice. J. Neuroinflammation.

[89] Paterniti I., Di Paola R., Campolo M., Siracusa R., Cordaro M., Bruschetta G., Tremolada G., Maestroni A., Bandello F., Esposito E. (2015). Palmitoylethanolamide treatment reduces retinal inflammation in streptozotocin-induced diabetic rats. Eur J. Pharm..

[90] Cordaro M., Impellizzeri D., Gugliandolo E., Siracusa R., Crupi R., Esposito E., Cuzzocrea S. (2016). Adelmidrol, a Palmitoylethanolamide Analogue, as a New Pharmacological Treatment for the Management of Inflammatory Bowel Disease. Mol. Pharm..

[91] Di Paola R., Cordaro M., Crupi R., Siracusa R., Campolo M., Bruschetta G., Fusco R., Pugliatti P., Esposito E., Cuzzocrea S. (2016). Protective Effects of Ultramicronized Palmitoylethanolamide (PEA-um) in Myocardial Ischaemia and Reperfusion Injury in VIVO. Shock.

[92] Di Paola R., Impellizzeri D., Fusco R., Cordaro M., Siracusa R., Crupi R., Esposito E., Cuzzocrea S. (2016). Ultramicronized palmitoylethanolamide (PEA-um((R))) in the treatment of idiopathic pulmonary fibrosis. Pharm. Res..

[93] Esposito E., Impellizzeri D., Bruschetta G., Cordaro M., Siracusa R., Gugliandolo E., Crupi R., Cuzzocrea S. (2016). A new co-micronized composite containing palmitoylethanolamide and polydatin shows superior oral efficacy compared to their association in a rat paw model of carrageenan-induced inflammation. Eur. J. Pharm..

[94] Impellizzeri D., Cordaro M., Bruschetta G., Crupi R., Pascali J., Alfonsi D., Marcolongo G., Cuzzocrea S. (2016). 2-pentadecyl-2-oxazoline: Identification in coffee, synthesis and activity in a rat model of carrageenan-induced hindpaw inflammation. Pharm. Res..

[95] Fusco R., D’Amico R., Cordaro M., Gugliandolo E., Siracusa R., Peritore A.F., Crupi R., Impellizzeri D., Cuzzocrea S., Di Paola R. (2018). Absence of formyl peptide receptor 1 causes endometriotic lesion regression in a mouse model of surgically-induced endometriosis. Oncotarget.

[96] Gugliandolo E., D’Amico R., Cordaro M., Fusco R., Siracusa R., Crupi R., Impellizzeri D., Cuzzocrea S., Di Paola R. (2018). Effect of PEA-OXA on neuropathic pain and functional recovery after sciatic nerve crush. J. Neuroinflam..

[97] Fusco R., Siracusa R., Peritore A.F., Gugliandolo E., Genovese T., D’Amico R., Cordaro M., Crupi R., Mandalari G., Impellizzeri D. (2020). The Role of Cashew (Anacardium occidentale L.) Nuts on an Experimental Model of Painful Degenerative Joint Disease. Antioxidants.

[98] Siracusa R., Fusco R., Peritore A.F., Cordaro M., D’Amico R., Genovese T., Gugliandolo E., Crupi R., Smeriglio A., Mandalari G. (2020). The Antioxidant and Anti-Inflammatory Properties of Anacardium occidentale L. Cashew Nuts in a Mouse Model of Colitis. Nutrients.

[99] Di Paola R., Fusco R., Impellizzeri D., Cordaro M., Britti D., Morittu V.M., Evangelista M., Cuzzocrea S. (2016). Adelmidrol, in combination with hyaluronic acid, displays increased anti-inflammatory and analgesic effects against monosodium iodoacetate-induced osteoarthritis in rats. Arthritis Res. Ther..

[100] Cuzzocrea S., Mazzon E., Esposito E., Muia C., Abdelrahman M., Di Paola R., Crisafulli C., Bramanti P., Thiemermann C. (2007). Glycogen synthase kinase-3beta inhibition attenuates the development of ischaemia/reperfusion injury of the gut. Intensive Care Med..

[101] Costantino G., Cuzzocrea S., Mazzon E., Caputi A.P. (1998). Protective effects of melatonin in zymosan-activated plasma-induced paw inflammation. Eur J. Pharmacol..

[102] Impellizzeri D., Esposito E., Di Paola R., Ahmad A., Campolo M., Peli A., Morittu V.M., Britti D., Cuzzocrea S. (2013). Palmitoylethanolamide and luteolin ameliorate development of arthritis caused by injection of collagen type II in mice. Arthritis Res. Ther..

[103] Di Paola R., Fusco R., Gugliandolo E., D’Amico R., Campolo M., Latteri S., Carughi A., Mandalari G., Cuzzocrea S. (2018). The Antioxidant Activity of Pistachios Reduces Cardiac Tissue Injury of Acute Ischemia/Reperfusion (I/R) in Diabetic Streptozotocin (STZ)-Induced Hyperglycaemic Rats. Front. Pharmacol..

[104] Cordaro M., Siracusa R., Impellizzeri D., D’ Amico R., Peritore A.F., Crupi R., Gugliandolo E., Fusco R., Di Paola R., Schievano C. (2019). Safety and efficacy of a new micronized formulation of the ALIAmide palmitoylglucosamine in preclinical models of inflammation and osteoarthritis pain. Arthritis Res. Ther..

[105] Fusco R., Siracusa R., D’Amico R., Peritore A.F., Cordaro M., Gugliandolo E., Crupi R., Impellizzeri D., Cuzzocrea S., Di Paola R. (2019). Melatonin Plus Folic Acid Treatment Ameliorates Reserpine-Induced Fibromyalgia: An Evaluation of Pain, Oxidative Stress, and Inflammation. Antioxidants.

[106] Liang J., Wu S., Xie W., He H. (2018). Ketamine ameliorates oxidative stress-induced apoptosis in experimental traumatic brain injury via the Nrf2 pathway. Drug Des. Devel Ther..

[107] Zhang R., Liu C., Li Y., Chen L., Xiang J. (2020). Tenacissoside H Promotes Neurological Recovery of Cerebral Ischemia-reperfusion Injury in Mice by Modulating Inflammation and Oxidative stress via TrkB Pathway. Clin. Exp. Pharm. Physiol..

[108] Shi D.D., Huang Y.H., Lai C.S.W., Dong C.M., Ho L.C., Wu E.X., Li Q., Wang X.M., Chung S.K., Sham P.C. (2019). Chemotherapy-Induced Cognitive Impairment Is Associated with Cytokine Dysregulation and Disruptions in Neuroplasticity. Mol. Neurobiol..

[109] Zhu N., Liang X., Zhang M., Yin X., Yang H., Zhi Y., Ying G., Zou J., Chen L., Yao X. (2020). Astaxanthin protects cognitive function of vascular dementia. Behav. Brain Funct..

[110] Peritore A.F., Siracusa R., Fusco R., Gugliandolo E., D’Amico R., Cordaro M., Crupi R., Genovese T., Impellizzeri D., Cuzzocrea S. (2020). Ultramicronized Palmitoylethanolamide and Paracetamol, a New Association to Relieve Hyperalgesia and Pain in a Sciatic Nerve Injury Model in Rat. Int. J. Mol. Sci..

[111] Loetchutinat C., Kothan S., Dechsupa S., Meesungnoen J., Jay-Gerin J.-P., Mankhetkorn S. (2005). Spectrofluorometric determination of intracellular levels of reactive oxygen species in drug-sensitive and drug-resistant cancer cells using the 2′, 7′-dichlorofluorescein diacetate assay. Radiat. Phys. Chem..

[112] Misra H.P., Fridovich I. (1972). The role of superoxide anion in the autoxidation of epinephrine and a simple assay for superoxide dismutase. J. Biol. Chem..

[113] Souza L.C., Antunes M.S., Filho C.B., Del Fabbro L., de Gomes M.G., Goes A.T., Donato F., Prigol M., Boeira S.P., Jesse C.R. (2015). Flavonoid Chrysin prevents age-related cognitive decline via attenuation of oxidative stress and modulation of BDNF levels in aged mouse brain. Pharm. Biochem. Behav..

[114] Wendel A. (1981). Detoxi¢ cation and drug metabolism; conjugation and related systems. Methods Enzym..

