# Supplementary figures and images for: Atrazine Inhalation Causes Neuroinflammation, Apoptosis and Accelerating Brain Aging
Source: Int J Mol Sci. 2021 Jul 26;22(15):7938. doi: 10.3390/ijms22157938 (PMC8347547; doi:10.3390/ijms22157938)

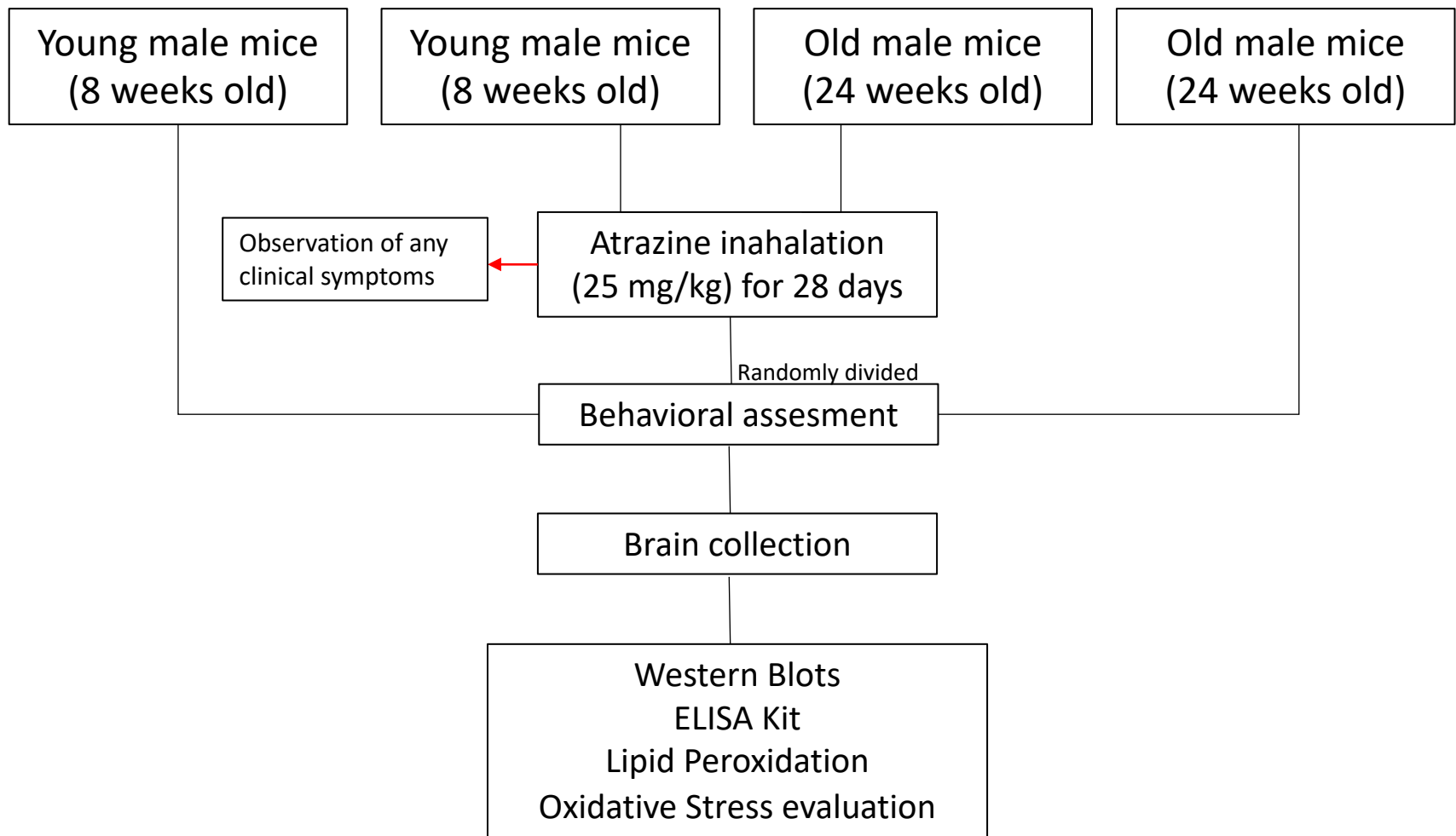

Supplement: Supplementary file 1 [file ijms-22-07938-s001.zip › ijms-1312149-supplementary.pdf]
